# Diversity of Endophytic Fungi in *Theobroma grandiflorum* and their Potential for Biological Control of Witches’ Broom Disease and Promotion of Cupuaçu Seedling Growth in the Amazon

**DOI:** 10.1007/s00284-026-04853-x

**Published:** 2026-04-03

**Authors:** Jusley Souza Santos, Nárcya Trindade de Souza, Thalya da Silva Rodrigues, Fernando José Fernandes Martins, Laryssa dos Santos Prado, Fernanda Viana Diniz, Erlangela Rocha Viga, Berenice Kussumoto de Alcântara da Silva, Clarice Maia Carvalho, Leila Priscila Peters

**Affiliations:** 1 Centro de Ciências Biológicas e da Natureza, Universidade Federal do Acre, Rio Branco, CEP, 69920-900 Acre Brazil; 2 Centro de Ciências da Saúde e do Desporto, Universidade Federal do Acre, Rio Branco, CEP, 69920-900 Acre Brazil

## Abstract

Endophytic fungi can play a crucial role in protecting plants against pathogens. Cupuaçu tree (*Theobroma grandiflorum*) is a commercially valuable crop significantly affected by witches’ broom, a disease caused by the pathogen *Moniliophthora perniciosa*. In the current study, we report the first investigation on the cultivable fungal endophytic community associated with the leaves and stems of *T. grandiflorum* in the Western Amazonia. We also selected 14 endophytic fungi to analyze hydrolytic enzymes and screen their antagonistic activity against *M. perniciosa* under laboratory and greenhouse conditions. Our findings revealed that the diversity and composition of the endophytic communities did not differ significantly between the sampled leaf and stem tissues. Most of the endophytic fungi exhibited amylolytic activity (93%), while 71% showed cellulolytic activity and 43% displayed proteolytic activity. Additionally, antagonism assays with *M. perniciosa* revealed that *Trichoderma orientale* showed the highest growth inhibition (69.4%), followed by *Cunninghamella blakesleeana* (64.2%) and *T. tawa* (63.6%). In greenhouse experiments, *T. tawa* and *C. blakesleeana* significantly reduced infection by *M. perniciosa* by 50% and 70%, respectively. Plants co-inoculated with *C. blakesleeana* and the pathogen showed a significant increase in height, shoot dry biomass, and photosynthetic pigment content compared to plants infected only with *M. perniciosa*. Furthermore, the application of *T. tawa* and *C. blakesleeana* increased the levels of chlorophylls and carotenoids in healthy plants, indicating a beneficial interaction with the plant’s physiology. Thus, these fungi stand out as promising candidates for the development of bioinputs aimed at the sustainable management of diseases in cupuaçu cultivation in the Amazon.

**Supplementary Information:**

The online version contains supplementary material available at 10.1007/s00284-026-04853-x.

## Introduction

Cupuaçu (*Theobroma grandiflorum*) is a fruit tree native to the Amazon region that belongs to the Malvaceae family, and is considered a domesticated variant of its wild relative, cupuí (*T. subincanum*) [1]. This species holds significant economic and social potential due to the versatile uses of its pulp and seeds, primarily in the food and cosmetic industries [2]. The pulp is used to produce a variety of products such as juices, ice creams, jellies, and cosmetics [3]. The seeds contain a fine, digestible fat, and can be processed into a product that is similar to chocolate, known as cupulate [4]. Additionally, cupuaçu cultivation is well-suited for agroforestry systems, which contribute to environmental conservation by providing an alternative to deforestation in the Amazon. This underscores its importance in the development of the bioeconomy of the Amazon biome [5].

However, cupuaçu plantations face significant challenges due to witches’ broom, one of the most severe diseases affecting this crop [6]. This disease drastically reduces yield, and consequently, farmer income [5], with losses of up to 90% in cupuaçu production [7]. Witches’ broom is caused by the basidiomycete phytopathogen *Moniliophthora perniciosa* [8]. This pathogen has two growth stages in the plant: the biotrophic phase and the necrotrophic phase [9]. In the initial biotrophic phase, the fungus penetrates the plant through meristematic tissues (such as shoots, young fruits, and floral pads), developing as a monokaryotic mycelium without connecting clamps. This results in the formation of a structure called green broom, which is caused by physiological and morphological changes induced by the fungus [10]. As the disease progresses, the fungus transitions to its necrotrophic phase, developing dikaryotic mycelium with connecting clamps. At this stage, *M. perniciosa* causes necrosis of infected tissues, leading to the development of dry broom and subsequent cell death [9–11].

The control of witches’ broom in cupuaçu trees is typically achieved through a combination of sanitary pruning and fungicides that inhibit ergosterol biosynthesis [12]. However, the use of synthetic pesticides has negative consequences for both terrestrial and aquatic environments and promotes the development of resistance mechanisms in pathogen populations [13–15]. Additionally, the rising cost of synthetic pesticides, coupled with consumer demand for xenobiotic-free food, has spurred the search for alternatives [16]. In this context, exploring new methods to protect plants from pathogens can significantly contribute to the sustainable cultivation of cupuaçu in the Amazon.

Alternative approaches are therefore being used to prevent plant diseases, with biological control of phytopathogens standing out among them [17, 18]. Often, this control can be achieved by endophytic fungi, which, by sharing the same habitat as the pathogens, become potential biocontrol agents [19, 20]. The ability of endophytic fungi to be effective biocontrol agents lies in their capacity to penetrate the plant and systemically colonize the host, competing for space with the phytopathogen [21]. Furthermore, they produce bioactive metabolites and enzymes that suppress diseases [22], aid in nutrient acquisition [23], promote plant growth [24], and increase tolerance to abiotic stresses [25].

Previous studies have demonstrated the ability of endophytic fungi to control *M. perniciosa in vitro* [26–29]. However, while several in vitro studies have shown promising results, in vivo experiments remain limited. In cacao plants (*T. cacao*), a species genetically related to the cupuaçu tree, some endophytic fungi were tested, but only *Gliocladium catenulatum* was able to successfully control witches’ broom disease *in planta* [26]. However, little is known about the endophytic fungi present in the cupuaçu tree and the ability of these microorganisms to control *M. perniciosa* in cupuaçu plants.

Therefore, the objectives of this study were: (i) to isolate endophytic fungi from healthy leaves and stems of cupuaçu in the southwestern Amazon; (ii) to compare the diversity of the cultivable endophytic fungal community between leaves and stems of cupuaçu; (iii) to evaluate in vitro the activity of hydrolytic enzymes and the antagonistic action of endophytic fungi against *M. perniciosa*; and (iv) to test the control of *M. perniciosa* by endophytic fungi in cupuaçu plants.

## Materials and Methods

### Sampling Locations

Our study investigated endophytic fungi isolated from *Theobroma grandiflorum* (Willd. ex Spreng.) K. Schum (cupuaçu). The sampling sites were located in the southwestern Amazon, specifically in the states of Acre and Rondônia, Brazil (Fig. S1). In Acre, cupuaçu trees were sampled in the municipalities of Rio Branco, Cruzeiro do Sul, Feijó, Porto Acre, Sena Madureira, Epitaciolândia and Brasiléia. In Rondônia, sampling was conducted in the municipality of Nova Califórnia, between March and May 2019.

At each location, we collected three leaves (from positions 3–5 on different lower branches of the canopy) and three branches per tree. In addition, we collected stem samples from the trees. For this, at a height of 50 cm from the ground, a fragment of the bark was removed from a branch to collect cortical tissue from the cupuaçu tree stem. The sampling site was then sealed with paraffin wax to prevent the entry of pathogens or pests. In each municipality (*n* = 8), leaves and stems were sampled from three trees, totaling 24 trees evaluated. The cupuaçu trees showed the following characteristics: they appeared healthy and were free from chemical or biological treatments. These trees were cultivated in agroforestry systems and were not part of any genetic improvement programs. Other species present in the agroforestry systems included açaí (*Euterpe precatoria*), peach palm (*Bactris gasipaes*), cacao (*Theobroma cacao*), and banana (*Musa* spp.). The biological material collected was stored separately in sterile transparent plastic bags at 4 °C and processed within 24 h [30].

### Isolation of Endophytic Fungi

#### Culture Media

In order to isolate different endophytic fungi two culture media were used: Potato Dextrose Agar – PDA (200 g potato extract, 20 g dextrose, 15 g agar, per L of distilled water, pH 6.5); Oatmeal Agar – OA (30 g oat flakes, 13 g agar, per L of distilled water, pH 6.5). To prepare PDA and OA media, potato and oat flakes extracts were heated in distilled water for 15 min. The infusion was filtered, with dextrose and agar added to prepare PDA medium, while only agar was added to OA medium. Both media were supplemented with chloramphenicol at 0.01% (w/v). Subsequently, the media were autoclaved at 121 °C for 20 min. In addition to these culture media, Niger Agar medium (NG) was also used for micromorphological analyses (50 g of sunflower seeds, 15 g of agar, per liter of distilled water, pH 6.5). The seeds were boiled in distilled water until the seed coats were removed, then ground and filtered. Distilled water and agar were added to the filtrate, followed by autoclaving at 121 °C for 20 min.

#### Isolation Procedures

Endophytic fungi were isolated from cupuaçu leaves and stems using the method described by Pereira et al. [31]. The sampled plant tissues were washed with running water and then superficially sterilized with 70% ethanol for 1 min, followed by a 3-minute immersion in 2.5% sodium hypochlorite (NaClO) solution, and then, rinsed three times with sterile distilled water. After sterilization, the bark of the cupuaçu branches was removed to access the stem, and 1-cm diameter fragments were obtained. For the leaves, 5 mm diameter fragments were used.

All fragments were placed separately in Petri dishes (90 × 15 mm) containing PDA and OA media, supplemented with 0.01% (w/v) chloramphenicol. The plates were incubated at 28 °C in the dark for 30 days. To check the efficiency of the surface sterilization method, 100 µL of distilled water from the last rinse was inoculated onto the PDA medium at 28 °C in the dark, and analyzed after 24 h [30].

Fungal isolates were purified by streaking on agar plates, where isolated colonies were selected to obtain pure cultures for morphological identification. Mycelial fragments from fungal colonies were inoculated into PDA medium, incubated at 28 °C in the dark, and analyzed after 14 days. Endophytic fungi were categorized into morphotypes based on colony morphology (color, texture, shape, and pigment production) and reproductive structures (hyphae and conidia characteristics) [30].

A representative isolate of each morphotype was cultivated on PDA medium at 28 °C in the dark for 14 days for microscopic identification [30]. Micromorphological analysis was performed using microculture techniques on PDA, Oatmeal Agar, and Niger Agar. Plates were incubated at room temperature for 14 days under a 12 h dark/12 h light cycle, and cover slips were stained with lactophenol blue to view reproductive structures [32, 33]. Endophytic fungi were stored in distilled water [34] and mineral oil [35] and deposited in the microorganism collection of the Laboratório de Microbiologia of Universidade Federal do Acre (UFAC), Brazil.

### Molecular Identification of Endophytic Fungal Isolates

Fungi that exhibited rapid growth at 28 °C in the dark, covering the entire surface of a Petri dish (90 × 15 mm) with mycelium within five days, and that sporulated on PDA medium were selected for molecular identification. These microorganisms were cultivated on PDA medium at 28 °C for 14 days in the dark. DNA was extracted using the Quick-DNA Fungal/Bacterial Miniprep Kit (Zymo Research) according to the manufacturer’s instructions. The rDNA ITS 5.8 S region was amplified using primers ITS1 and ITS4 [36].

For amplification, a PCR reaction (25 µL) was prepared using 2 µL of DNA (20 ng), 1 U of Taq DNA Polymerase (Ludwig), 0.5 µL of each primer (10 µM), 0.75 µL of MgCl₂, 0.5 µL of dNTPs, 2.5 µL of 1x buffer, and the total volume was adjusted with Milli-Q water. The thermocycler (Bio-Rad T100) was programmed for an initial denaturation at 95 °C for 2 min, followed by 35 cycles of amplification (95 °C for 30 s, 55 °C for 30 s, and 72 °C for 1 min), and a final extension at 72 °C for 7 min, maintaining at 12 °C. PCR products were purified using the PCR Purification Kit (Mebep Bioscience) and quantified on a 2% agarose gel stained with SYBR^®^ Safe DNA Gel Stain for 45 min at 120 V with a molecular marker (Invitrogen DNA 1 kb Plus Ladder) of 100 bp.

Forward and reverse sequencing reactions were performed on a 7330xl DNA analyzer (Applied Biosystems). Nucleotide sequences were edited using Chromas (http://www.technelysium.com.au/chromas.html*).* All sequences were manually checked, and nucleotides with ambiguous positions were edited. Consensus sequences were assembled using BioEdit version v 7.0.5 [37]. Sequences were analyzed using BLASTn at NCBI (The National Center for Biotechnology Information) for fungal identification [38]. The sequences were deposited in the NCBI GenBank database (https://www.ncbi.nlm.nih.gov/nuccore/PQ900713*)* (Table 2).

**Table 1:** Taxonomic classification and relative frequency of endophytic fungi recovered from cupuaçu leaves and stems

Phylum	Class	Order	Family	Genera	Leaf	Stem	Total	RF* (%)
Ascomycota (90.60%)	Sordariomycetes (58.2%)	Diaporthales	Diaporthaceae	*Diaporthe*	101	52	153	31.81
		Glomerellales	Glomerellaceae	*Colletotrichum*	31	8	39	8.11
		Xylariales	Xylariaceae	*Xylaria*	12	11	23	4.78
		Hypocreales	Nectriaceae	*Fusarium*	6	10	16	3.33
		Xylariales	Amphisphaeriaceae	*Pestalotiopsis*	11	5	16	3.33
		Hypocreales	Hypocreaceae	*Trichoderma*	0	12	12	2.49
		Amphisphaeriales	Pestalotiopsidaceae	*Neopestalotiopsis*	2	3	5	1.04
		Xylariales	Xylariaceae	*Daldinia*	0	5	5	1.04
		Hypocreales	Bionectriaceae	*Clonostachys*	2	1	3	0.62
		Xylariales	Xylariaceae	*Hypoxylon*	3	0	3	0.62
		Sordariales	Sordariaceae	*Gelasinospora*	0	2	2	0.42
		Incertae sedis	Incertae sedis	*Myrmecridium*	0	2	2	0.42
		Trichosphaeriales	Trichosphaeriaceae	*Nigrospora*	1	0	1	0.21
	Dothideomycetes (25.2%)	Botryosphaeriales	Botryosphaeriaceae	*Lasiodiplodia*	42	48	90	18.71
		Botryosphaeriales	Botryosphaeriaceae	*Guignardia*	20	0	20	4.16
		Pleosporales	Pleosporaceae	*Curvularia*	0	5	5	1.04
		Botryosphaeriales	Endomelanconiopsidaceae	*Endomelanconiopsis*	0	3	3	0.62
		Capnodiales	Cladosporiaceae	*Cladosporium*	2	1	3	0.62
	Eurotiomycetes (6%)	Eurotiales	Aspergillaceae	*Penicillium*	13	11	24	4.99
		Eurotiales	Trichocomaceae	*Talaromyces*	3	2	5	1.04
	Saccharomycetes (1.2%)	Saccharomycetales	Dipodascaceae	*Geotrichum*	6	0	6	1.25
Basidiomycota (0.21%)	Agaricomycetes (0.21%)	Agaricales	Psathyrellaceae	*Coprinellus*	0	1	1	0.21
Mucoromycota (0.21%)	Mucoromycetes (0.21%)	Mucorales	Cunninghamellaceae	*Cunninghamella*	1	0	1	0.21
				Micelya sterilia	25	18	43	8.94
				Total	281	200	481	100

*RF: relative frequency

### Assessment of Endophytic Fungal Community Diversity

To evaluate the diversity of isolated endophytic fungal communities, parameters such as relative abundance and species richness were used. Diversity was assessed using two indices: the Shannon-Weaver index [39] and the Simpson index [40]. To compare diversity between leaves and stems, Hutcheson’s t-test was applied for the Shannon-Weaver index [41], and Student’s t-test was used for Simpson’s index, evenness, abundance, and richness. Analyses were performed using PAST version 4.03 (https://past.en.lo4d.com/windows*).*

Non-metric multidimensional scaling (NMDS) was used to evaluate the similarity of fungal communities in leaves and stems. Additionally, an Analysis of Similarity (ANOSIM) tested for significant differences (*p* < 0.05) between fungal community clusters obtained from NMDS ordination, using Bray-Curtis distance matrices. This analysis compared species composition between groups (leaves and stems) and generated an R value indicating the degree of discrimination between groups. Analyses were conducted using RStudio.

### Analysis of Extracellular Enzymes

For enzymatic analysis, 14 endophytic fungi with good growth and sporulation on PDA medium were selected. These fungi were cultivated on PDA medium at 28 °C for 14 days in the dark. Mycelium discs (5 mm in diameter) were removed and inoculated into specific culture media for each extracellular enzyme assessed. The enzymatic index (EI) was calculated as the ratio of the diameter of the degradation zone to the diameter of the fungal colony [42]. For each enzyme, 5 replicates were performed (*n* = 5). The diameter of the halos and the fungal colonies was measured in centimeters with a millimeter-scale ruler.

Amylolytic activity was assessed using M9 minimal medium (0.19 g NaNO₃, 0.25 g KCl, 0.59 g KH₂PO₄, 0.25 g MgSO₄·7 H₂O, 0.005 g FeSO₄·7 H₂O, 15 g agar, pH 6.0) with 2.7 g meat extract, 4.5 g peptone, and 1% starch. After 7 days at 28 °C in the dark, an iodine solution was used to reveal translucent halos around colonies that were indicative of starch degradation [43]. Cellulose degradation was tested by inoculating 5-mm mycelial discs in a medium containing 15 g agar and 1% carboxymethylcellulose (CMC), and incubating at 28 °C for 7 days in the dark [44]. Plates were stained to evaluate cellulase activity. Lipid degradation was evaluated by inoculating fungi into minimal medium (MM; 0.19 g NaNO₃, 0.25 g KCl, 0.59 g KH₂PO₄, 0.25 g MgSO₄·7 H₂O, 0.005 g FeSO₄·7 H₂O, 5 g glucose, 15 g agar, pH 6.5) supplemented with 2% Tween 80. Positive reactions were indicated by lauric acid crystals around colonies after 7 days at 28 °C and an additional 7 days at 4 °C [42]. Proteolytic activity was analyzed using minimal medium supplemented with 2% skim milk (pH 6.5). Positive reactions were indicated by translucent halos around colonies [45].

### In Vitro Antagonism Assays between Endophytic Fungi and *M. perniciosa*

Dual assays were performed to evaluate the antagonistic activity between the 14 endophytic fungi and *M. perniciosa*. The pathogen was isolated from basidiomata collected from dried witches’ brooms of *T. grandiflorum* in Nova Califórnia, RO (Brazil). Basidiomata were sterilized in 3% sodium hypochlorite (NaClO) for 3 min, and then, cut into five fragments. The fragments were placed in Petri dishes with PDA medium, supplemented with 0.01% (w/v) chloramphenicol and 0.01% (w/v) benomyl, and incubated at 28 °C for 14 days [30].

Mycelium discs (5 mm in diameter) from fungal colonies were placed in 9-cm Petri dishes with PDA medium, 3 cm away from the pathogen disc. *M. perniciosa* was inoculated 5 days before endophytic fungi [26]. Control plates contained only the pathogen. Plates were incubated at 28 °C for 14 days, with three replicates per treatment [46, 47]. The radial growth of *M. perniciosa* colonies was measured on day 14. The inhibition coefficient (IC%) was calculated as follows: IC% = [(CC - DC) / CC] × 100, where CC is the radial growth of *M. perniciosa* in the control culture and DC is the growth in culture with endophytic fungi [48]. Additionally, the macroscopic interaction between the pathogen and endophytic fungi was classified into four categories: (1) contact inhibition, characterized by the cessation of growth of both organisms at the point of contact, without the formation of a clear zone; (2) inhibition at a distance, in which neither organism advances into the area occupied by the other, resulting in a visible clear zone; (3) overgrowth, when the mycelium of one species extends over the other; and (4) replacement, where the mycelium of one fungus is substituted by that of its antagonist [47].

### *In planta* Antagonism Test between Endophytic Fungi and *M. perniciosa*

Two experiments were conducted in a completely randomized design in a greenhouse. The first experiment screened 14 endophytic fungi for activity against *M. perniciosa* in cupuaçu seedlings. In this first in vivo antagonism assay, the endophytic fungi were selected based on their rapid growth and ability to sporulate on PDA medium. The second experiment was carried out with the two fungi that showed the best results in controlling the pathogen *in planta* (according to the results from the first experiment). In both experiments, the treatments included: plants inoculated with endophyte (1), plants inoculated with endophyte and pathogen (2), plants inoculated only with the pathogen (positive control, 3), and plants inoculated with a suspension without fungal spores (negative control, 4). In the first experiment, disease incidence, plant height (cm), and dry mass of leaves, stem, and root (g) were evaluated for 6 plants per treatment. In the second experiment, chlorophyll and carotenoid contents were also analyzed, with measurements recorded from 12 plants per treatment.

#### Cultivation of Cupuaçu Seedlings

Cupuaçu seeds (susceptible genotype) were obtained from healthy plants of an agroforestry system in Nova Califórnia, Rondônia, Brazil. Seeds were disinfected in a 1% (v/v) sodium hypochlorite solution for 1 min, and then, washed in distilled water for 2 min. Seeds were sown in polyethylene trays, covered with autoclaved sawdust, and kept in a greenhouse with a 10-hour light and 14-hour dark photoperiod, with temperatures of 32 ± 2 °C during the day and 25 ± 2 °C at night. After 20 days, seedlings were transferred to 3-L pots containing a soil-sand mixture (1:1:1 ratio), autoclaved for 60 min. Seedlings were kept in a greenhouse with 50% shade and were irrigated daily.

#### Inoculation of Endophytes and Pathogen

Endophytes were inoculated into 3-month-old seedlings. For each endophyte, a suspension of 10^6 spores/mL (containing 0.01% Tween 20) was prepared by adding sterilized distilled water to each plate containing the fungus grown in PDA or oat medium. Spore concentration was determined using a Nikon microscope (Nikon E200) and a hemocytometer. Endophytic fungal suspensions were sprayed onto seedling leaves with a hand sprayer. For 24 h post-inoculation, relative humidity was maintained close to 100% using plastic bags to stimulate spore germination [49]. Endophytic fungi were applied twice: 15 days before pathogen application and 1 day before. *M. perniciosa* basidiospores were obtained as described by Frias [50], and 30 µL of a 10^5-spores/mL suspension were inoculated into the apical meristem of seedlings [51].

#### Assessment of Disease and Growth of Cupuaçu Seedlings

Disease incidence (%) was determined at 90 days after inoculation (DAI) using the formula: (number of diseased plants / total number of evaluated plants) × 100 [52]. The plant growth parameters measured included height (cm) and dry mass of shoot and root (g). Plants were dried at 45 °C for 72 h and weighed on an analytical balance.

#### Chlorophyll and Carotenoid Content

Chlorophyll and carotenoid contents were analyzed for plants in the second experiment. To determine chlorophyll and carotenoid levels, 50 mg of leaves from each treatment were macerated and placed in glass tubes wrapped in aluminum foil containing 7 mL of 80% acetone solution. Tubes were stored at 4 °C for 48 h to extract photosynthetic pigments. Optical density was measured at 663, 645, and 470 nm wavelengths for chlorophyll a, b, and carotenoids, respectively. Levels were calculated according to the equations described in Lichtenthaler [53]:

Total chlorophyll = (17.3 × A647 + 7.18 × A663).

Chlorophyll a = (12.21 × A663–2.81 × A647).

Chlorophyll b = (20.13 × A647–5.03 × A663).

Carotenoids = [1000 × A470–3.27 × (chlorophyll a) − 104 × (chlorophyll b) / 229]

Results were expressed in mg/L and converted to mg/g of fresh mass [54].

### Statistical Analyses

In vitro and in vivo antagonism tests were performed for normality and homoscedasticity. Normality was evaluated using the Shapiro-Wilk test, and homogeneity of variances was assessed with the Bartlett test. Data significance was analyzed using one-way ANOVA followed by Tukey’s test (*p* < 0.05). Analyses were performed using software R. Principal component analysis (PCA) was used to compare plant growth parameters and disease symptoms between treatments.

## Results

### Culturable Endophytic Fungal Community

**Fig. 1:**
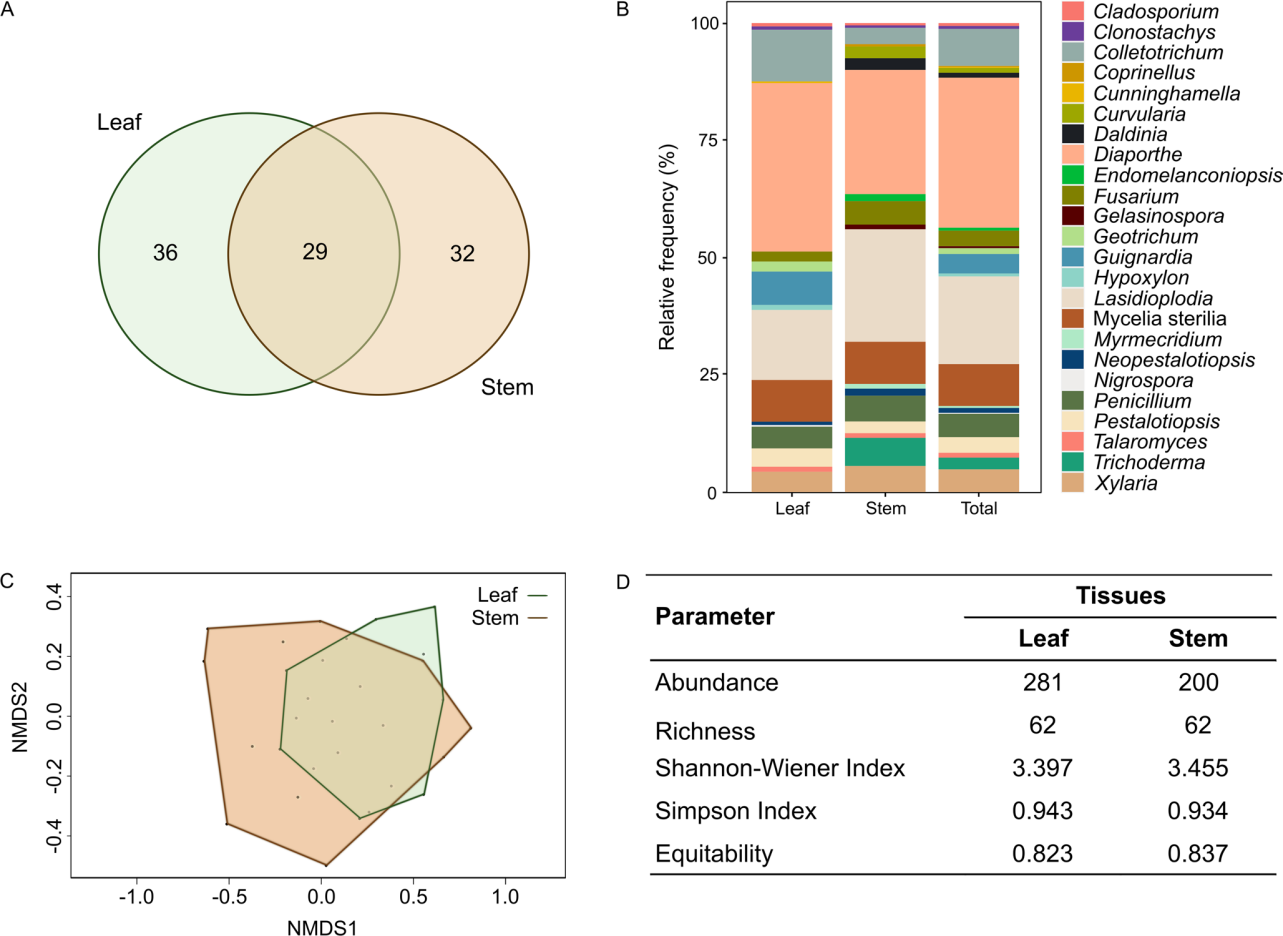
Comparison of endophytic fungi recovered from leaves and stem of *Theobroma grandiflorum.* **a** Venn diagram showing the distribution of fungal morphotypes in the studied communities. **b** Relative abundance of endophytic fungal genera. **c** Non-metric multidimensional scaling (nMDS) of endophytic fungal communities isolated from cupuaçu leaf and stem tissue based on morphotype level, Stress = 2,6. **d** Diversity parameters and descriptive data of the fungal communities recovered from cupuaçu leaf and stem tissue based on morphotype level

A total of 481 culturable endophytic fungi were recovered from *T. grandiflorum.* Leaf tissue harbored the highest number of fungal isolates, totaling 281, while the stem hosted 200 isolates (Table 1). The morphology of fungal colonies of several endophytic fungi from *T. grandiflorum* are shown in Figure S2. These endophytic fungi were grouped into 97 distinct morphotypes according to morphological characteristics. Of these morphotypes, 36 were recovered from leaves, 32 were isolated from stem tissue samples, and 29 were shared by both tissue types (Fig. 1a).

**Table 2:** Maximum nucleotide identity for 14 fungal isolates from cupuaçu tree was determined by analyzing ITS sequences through BLASTn

Fungi No.	GenBank Accession No.	DNA fragment size (pb)	Species identification based on closest GenBank match (GeneBank accession number)	Identity (%)	Plant tissue
2.6610	PQ900711	543	*Colletotrichum fructicola* (OQ652354)	100	Leaf
2.6184	PQ900712	596	*Trichoderma tawa* (KC847172)	100	Stem
2.6507	PQ900713	557	*Daldinia eschscholtzii* (MZ270647)	99.82	Stem
2.6685	PQ900714	631	*Cunninghamella blakesleeana* (NR_119974)	98.73	Leaf
2.6663	PQ900715	612	*Coprinellus radians* (MK843952)	99.84	Stem
2.6566	PQ900717	569	*Clonostachys rosea* (MT845995)	99.47	Stem
2.6238	PQ900718	560	*Clonostachys* sp. (KP998525)	100	Leaf
2.6694	PQ900719	557	*Myrmecridium* sp. (MT325794)	98.20	Stem
2.6107	PQ900720	633	*Trichoderma orientale* (PQ496846)	99.68	Stem
2.6182	PQ900721	572	*Talaromyces pinophilus* (MF972903)	100	Leaf
2.6240	PQ900722	550	*Talaromyces purpureogenus* (MK849928)	100	Leaf
2.6315	PQ900723	692	*Cladosporium* sp. (JX243752)	100	Stem
2.6690	PQ900993	557	*Gelasinospora calospora* (MN341352)	100	Stem
2.6265	PQ900994	481	*Endomelanconiopsis microspora* (MK371761)	100	Stem

*E value = 0.00 for all the results

The endophytic mycobiota isolated from leaf and stem tissues of *T. grandiflorum* predominantly comprises Ascomycota species (90.6%), which included four classes. The most frequent classes were Sordariomycetes (58.2%) and Dothideomycetes (25.2%), followed by Eurotiomycetes (6.0%) and Saccharomycetes (1.2%). These different classes were classified into 13 orders, and Diaporthales (31.8%) was the most frequent, followed by Botryosphaeriales (23.4%), Xylariales (9.7%), and Glomerellales (8.1%). The genera with the highest number of species were *Diaporthe* (31.8%), *Lasiodiplodia* (18.7%), *Colletotrichum* (8.1%), *Penicillium* (4.9%), and *Xylaria* (4.7%) (Table 1). The phylum Basidiomycota accounted for only 0.21% of the fungi isolated from *T. grandiflorum*, including the class Agaricomycetes, order Agaricales, and genus *Coprinellus*. Similarly, the phylum Mucoromycota, represented by the class Mucoromycetes, order Mucorales, and genus *Cunninghamella*, accounted for 0.21% of the fungal isolates recovered from cupuaçu trees (Table 1).

### Comparison of Fungal Endophytic Community from Leaves and Stems of *Theobroma grandiflorum*

Regarding relative abundance, the most common genera were *Diaporthe* and *Lasiodiplodia*, with 35.9% and 14.9% on cupuaçu leaves, respectively, and 26% and 24% in the stem tissue (Fig. 1b). The genera *Hypoxylon*, *Nigrospora*, *Geotrichum*, *Guignardia*, and *Cunninghamella* were recovered exclusively from cupuaçu tree leaves. On the other hand, the genera *Coprinellus*, *Gelasinospora*, *Daldinia*, *Myrmecridium*, *Trichoderma*, *Curvularia*, and *Endomelanconiopsis* occurred exclusively in the stem. Although the stem and leaf tissues had exclusive genera, the Shannon and Simpson indices and equitability did not show significant differences between the fungal endophytic community of cupuaçu leaves and that of the stem (Fig. 1d). Additionally, the non-metric multidimensional scaling (NMDS) plot revealed that the endophytic community composition did not differ significantly between the leaf and stem tissues sampled (Fig. 1c).

### Extracellular Enzyme Activities

Fourteen endophytic fungi isolated from *T. grandiflorum* were selected to qualitatively evaluate the activity of the enzymes amylase, cellulase, lipase, and protease. These fungi were selected because they grow easily in PDA medium and sporulate in large quantities in Oat or PDA medium. The identification of these fungi at the species level was carried out using the Blastn algorithm and is shown in Table 2.

**Fig. 2:**
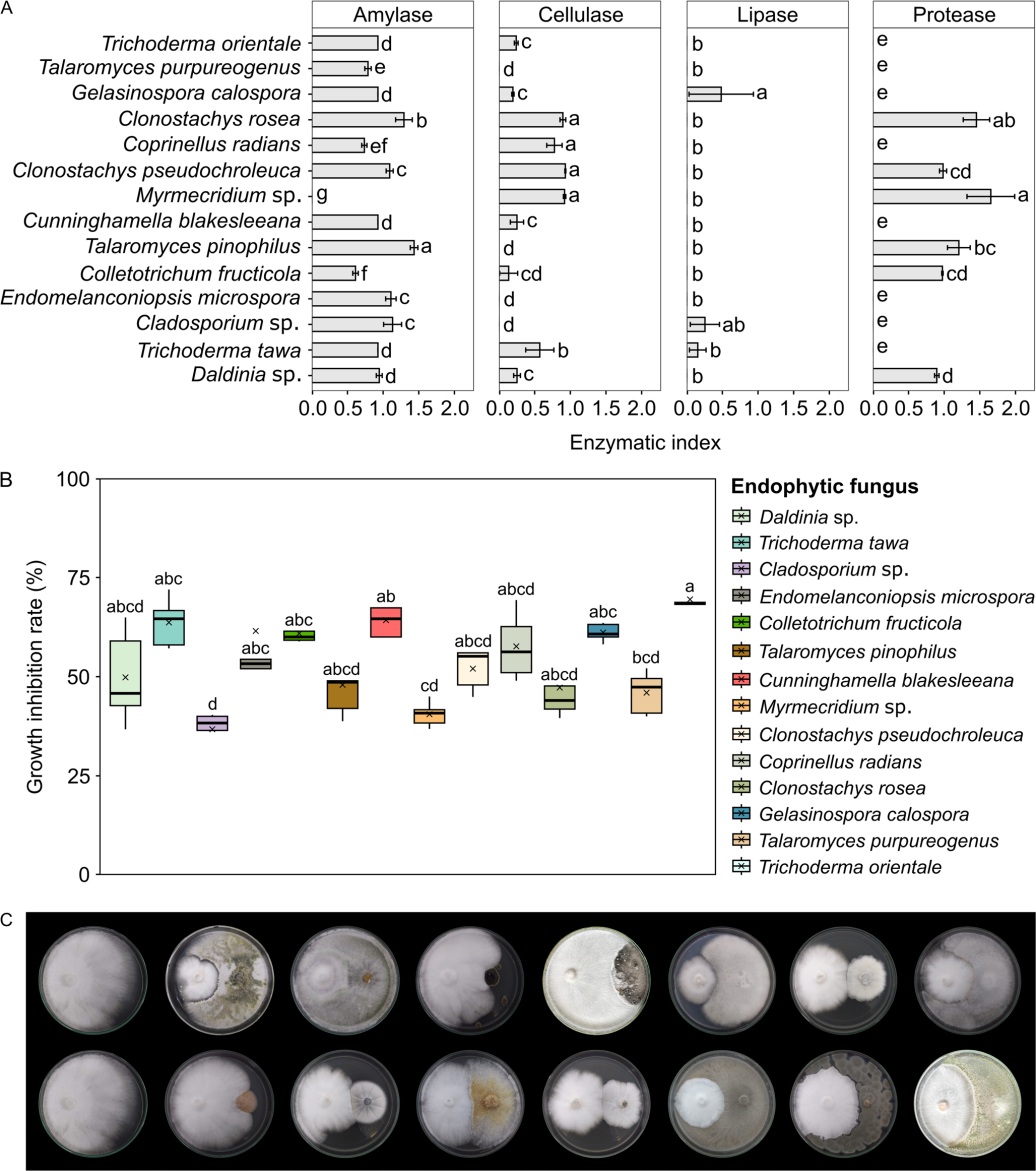
Enzymatic activities of endophytic fungi from *Theobroma grandiflorum* and their antagonistic activity against *Moniliophthora perniciosa*. **a** Enzymatic indices of the fungal isolates analyzed. **b** Antagonism assay evaluated between the endophytic fungi and the pathogen *M. perniciosa*. Data are presented as mean ± standard deviation of five biological replicates. Means with different letters are significantly different (*P* < 0.05) by one-way analysis of variance (ANOVA) and Tukey’s test. **c** Plates showing antagonism activity of the fungal endophytic isolates against *M. perniciosa*. The first row includes *Moniliophthora perniciosa* (control); *Daldinia* sp.; *Trichoderma tawa*; *Cladosporium* sp.; *Endomelanconiopsis microspora*; *Colletotrichum fructicola*; *Talaromyces pinophilus*; *Cunninghamella blakesleeana*, respectively. The second row includes *Moniliophthora perniciosa* (control); *Myrmecridium* sp.; *Clonostachys pseudochroleuca*; *Coprinellus radians*; *Clonostachys rosea*; *Gelasinospora calospora*; *Talaromyces purpureogenus*; *Trichoderma orientale*. *M. perniciosa* was inoculated on the right side of the plate, and the endophytic fungi were inoculated on the left side

All fungi exhibited amylase activity (93%) except for the *Myrmecridium* sp. isolate (Fig. 2a). The highest enzymatic indices for amylase production were found in *Talaromyces pinophilus*, followed by *Clonostachys rosea* and *C. pseudochroleuca* (Fig. 2a). Ten isolates (71%) of the fungal endophytes evaluated exhibited cellulase activity; C. *rosea*, *C. pseudochroleuca*, *Myrmecridium* sp., and *Coprinellus radians* had the highest enzymatic indices for cellulase (Fig. 2a). Six endophytic fungi (43%) showed positive results for proteolytic activity, including *Myrmecridium* sp., *C. rosea*,* T. pinophilus*,* C. pseudochroleuca*, and *Daldinia* sp. (Fig. 2a). Conversely, only 3 of the 14 fungi tested exhibited lipolytic activity (21%); *Gelasinospora calospora*,* Cladosporium* sp., and *Trichoderma tawa* (Fig. 2a). Endophytic fungi that produced a wider range of extracellular enzymes were *G. calospora*, *C. rosea*, *C. pseudochroleuca*, *Colletotrichum fructicola*, *T. tawa*, and *Daldinia* sp., which produced 3 types of extracellular enzymes evaluated.

### In Vitro Antagonism Assays between Endophytic Fungi and *M. perniciosa*

The antagonism assays between the 14 endophytic fungi and the pathogen *M. perniciosa* were expressed as percentage of growth inhibition (GI%) (Fig. 2b, c). *Trichoderma orientale* had the highest GI%, at 69.4%, and *Cunninghamella blakesleeana* had the second highest GI%, at 64.2%, followed by *T. tawa* (63.6%), *Endomelanconiopsis microspora* (61.5%), *Gelasinospora calospora* (61.1%), and *Colletotrichum fructicula* (60.8%). *Coprinellus radians and C. pseudochroleuca* had GI% of up to 57.6% and up to 52.0%, respectively. The other fungi evaluated had GI% below 50% (Fig. 2b, c). We observed a significant difference between the fungal isolates with the highest GI% (*T. orientale*, *C. blakesleeana*, and *T. tawa*) and the fungi with the lowest GI%, including *Cladosporium sp*., *Myrmecridium sp*., and *Talaromyces purpureogenus* (Fig. 2b, c).

Furthermore, the dual culture assay revealed that the main interaction between the endophytic isolates of *T. grandiflorum* and *M. perniciosa* was contact inhibition (*C. radians*, *Myrmecridium* sp., *G. calospora*, *T. pinophilus*, *Cladosporium* sp. *C. blakesleeana*, and *Daldinia* sp.). *C. rosea*, *C. fructicola*, and *C. pseudochroleuca* exhibited distant inhibition against *M. perniciosa*. Furthermore, the fungus *T. tawa* grew on the *M. perniciosa* colony (Fig. 2b, c).

### In Vivo Screening for Biocontrol Efficacy and Promotion of the Growth of Cupuaçu Seedlings

In the first experiment, 14 endophytic fungi were tested on cupuaçu plants under greenhouse conditions, both in the absence and presence of *M. perniciosa*. The disease incidence (%), dry weight of the shoot and root, and plant height for each treatment are shown in Table S1. After 3 months, cupuaçu plants inoculated with the pathogen did not exhibit disease symptoms when co-inoculated with the endophytic fungi *T. tawa*, *C. blakesleeana*, and *Daldinia* sp. (*p* < 0.05) (Table S1). Disease incidence in plants co-inoculated with the pathogen and the endophytic fungi *Cladosporium* sp., *Endomelanconiopsis microspora*, *Talaromyces pinophilus*, *Myrmecridium* sp., *Clonostachys pseudochroleuca*, *Coprinellus radians*, *C. rosea*, *Gelasinospora calospora*, *T. purpureogenus*, and *T. orientale* ranged from 17% to 67%. In contrast, the endophytic fungus *Colletotrichum fructicola* failed to control witches’ broom disease in cupuaçu seedlings. Furthermore, the endophytic fungi *C. blakesleeana* and *Daldinia* sp. significantly promoted shoot growth in cupuaçu seedlings even in the presence of *M. perniciosa* compared to both positive and negative controls (*p* < 0.05) (Table S1).

**Fig. 3:**
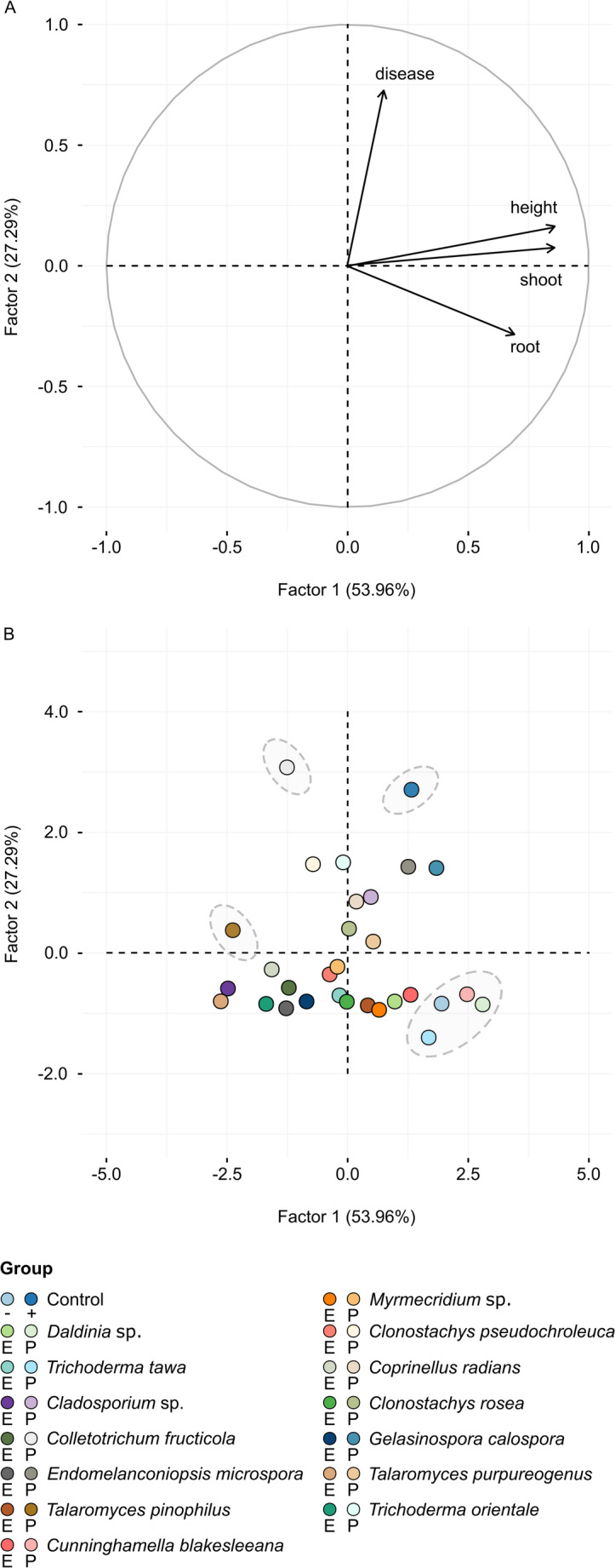
Principal component analysis (PCA) of plant growth parameters and disease incidence (%) in cupuaçu plants inoculated with each of the 14 fungal isolates, with and without *Moniliophthora perniciosa*, under greenhouse conditions. **a** Loading plot of plant growth and disease symptom parameters for the first two principle components, PC1 and PC2. **b** PCA (first two axes) showing groups of fungal isolates (dashed line circles) in plants inoculated only with endophytic fungi and co-inoculated with endophytic fungi and *M. perniciosa*. The letter *E* represents cupuaçu plants inoculated only with the endophytic fungus, while the letter *P* represents cupuaçu plants co-inoculated with the endophytic fungus and the pathogen

Figure 3a shows the loading plot of plant growth and disease incidence parameters for the first two principal components (PC1 and PC2), which together explained 81.25% of the total variance (PC1: 53.96%; PC2: 27.29%). PC1 was strongly associated with growth parameters (plant height, shoot, and roots), while PC2 reflected disease incidence. In Fig. 3b, the PCA showed the distribution of fungal isolate groups (represented by dashed circles) in plants inoculated only with endophytic fungi (E) and in plants co-inoculated with endophytic fungi and *M. perniciosa* (P). We observed that plants co-inoculated with the pathogen and the endophytic fungi *T. tawa*, *C. blakesleeana*, and *Daldinia* sp. tended to cluster with the negative control. In contrast, plants co-inoculated with the pathogen and *C. fructicola* showed results similar to those of the positive control (Fig. 3b).

### Validation of in Vivo Screening Results

**Fig. 4:**
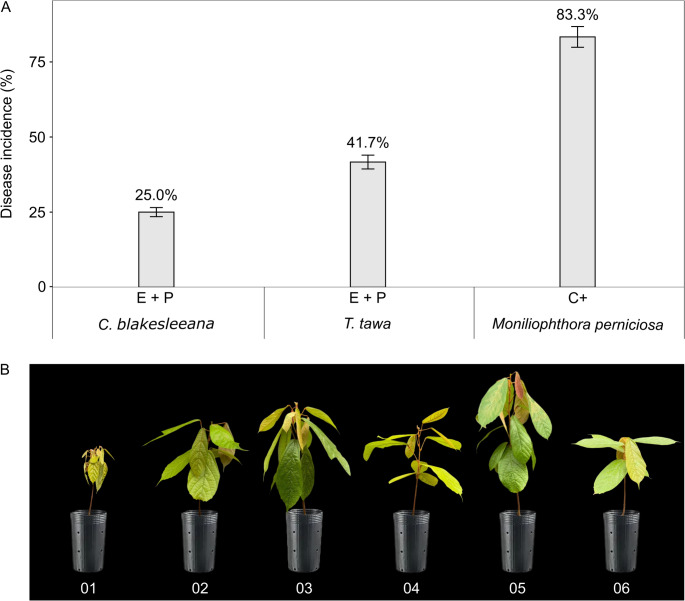
Disease incidence caused by *Moniliophthora perniciosa* in cupuaçu seedlings co-inoculated with the endophytic fungi *Cunninghamella blakesleeana* and *Trichoderma tawa*. **a** Disease incidence (%) in cupuaçu seedlings, E represents the endophytic fungus, P represents pathogen and C+ represents positive control (plants inoculated solely with *M. perniciosa*). Data are presented as mean ± standard deviation of 12 biological replicates. **b** Cupuaçu seedlings inoculated with endophytic fungus and control plants. 01. Positive control (plants inoculated only with pathogen). 02. Negative control (Mock-inoculated plant). 03. Plants inoculated with *T. tawa*. 04. Plants co-inoculated with *T. tawa* and *M. perniciosa*. 05. Plants inoculated with *C. blakesleeana*. 06. Plants co-inoculated with *C. blakesleeana* and *M. perniciosa*

**Fig. 5:**
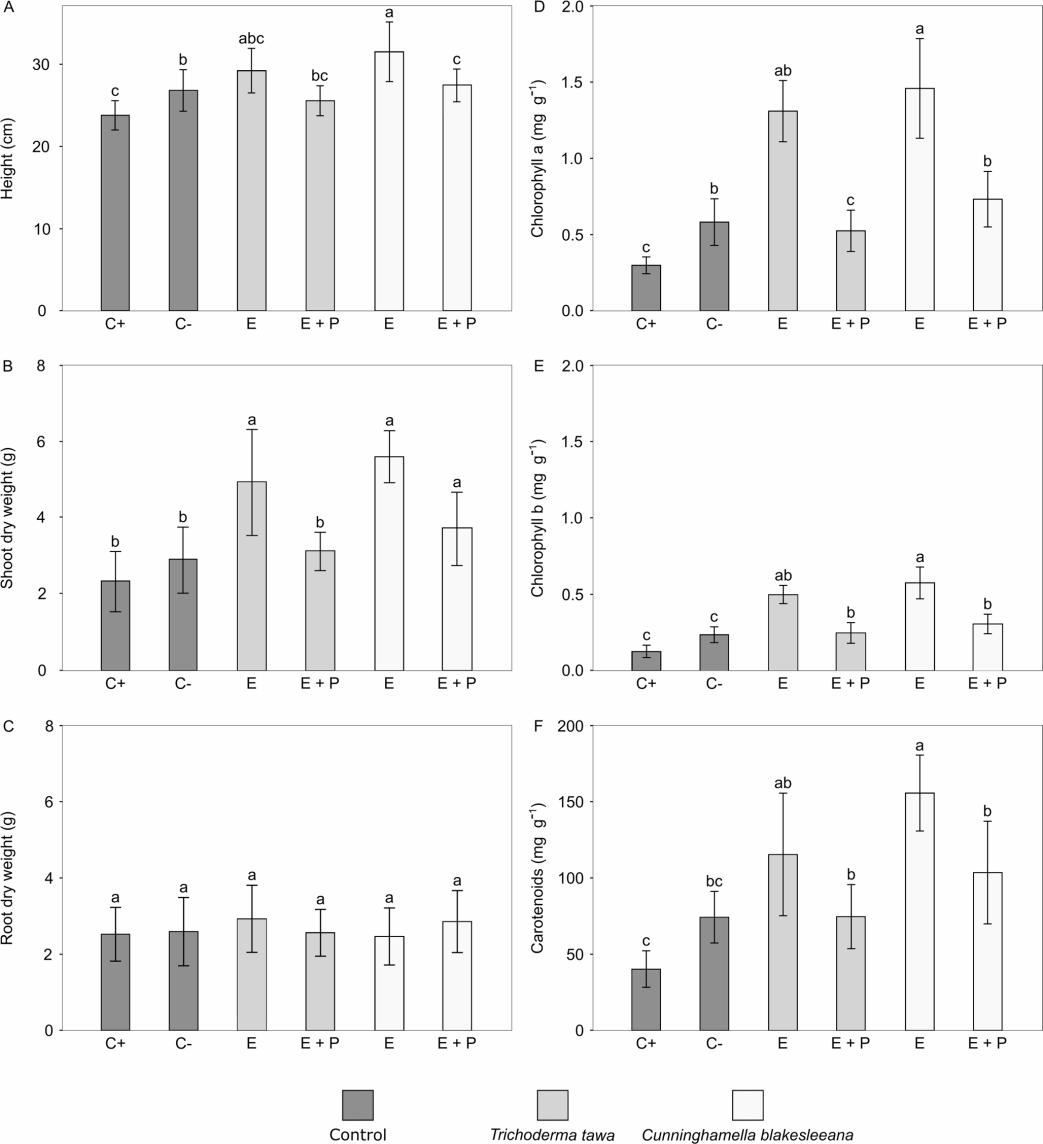
Effect of *Trichoderma tawa* and *Cunninghamella blakesleeana* inoculation in the presence of *Moniliophthora perniciosa* on the growth of cupuaçu seedlings and the synthesis of photosynthetic pigments. **a** Heights of cupuaçu seedlings. **b** Shoot dry weight. **c** Roots dry weight. **d** Chlorophyll *a*. **e** Chlorophyll *b*. **f** Carotenoids. E represents plant inoculated with endophytic fungus, E + P represents plants co-inoculated with the endophytic fungus and the pathogen, and C- and C+ represent negative and positive control, respectively. Data are presented as mean ± standard deviation of 12 biological replicates. Means with different letters are significantly different (*P* < 0.05) by one-way analysis of variance (ANOVA) and Tukey’s test

Two endophytic fungi were tested again to evaluate their efficiency in controlling *M. perniciosa* in cupuaçu plants under greenhouse conditions. *T. tawa* and *C. blakesleeana* were selected because their results were similar to the negative control when co-inoculated with the pathogen. Although *Daldinia eschscholtzii* showed results in controlling *M. perniciosa* in the first experiment, this endophyte fungus was unable to sporulate sufficiently in the culture medium. In the second experiment, the results showed that *T. tawa* and *C. blakesleeana* applied to cupuaçu plants could significantly reduce *M. perniciosa* infection by 50% and 70%, respectively, compared with plants infected only with the pathogen. The incidence of the disease in plants inoculated with *C. blaskesleeana* was 25% and in plants with *T. tawa*, it was 41% (Fig. 4a, b). Furthermore, we observed that the endophytic fungus *C. blakesleeana* caused a significant increase in the height and dry weight of the shoots of plants infected with *M. perniciosa*, compared to only infection with the pathogenic fungus (Fig. 5a, b). Additionally, treatments with only endophytic fungi exhibited significant increase in shoot growth and dry weight compared to the negative control. On the other hand, endophytic fungi did not alter root growth (Fig. 5c).

*M. perniciosa* infection in cupuaçu plants without the inoculation of endophytes caused a deficiency in photosynthetic pigments compared to the negative control, with Chl *a* and Chl *b* content decreasing by 48.7% and 45.1%, respectively (Fig. 5d, e). Conversely, the inoculation of the endophytes *T. tawa* and *C. blakesleeana* in cupuaçu plants resulted in a significant increase in the content of chlorophyll pigments, including Chl *a* (55.6% and 60.1%, respectively), Chl *b* (59.8% and 53.5%, respectively), and carotenoids (52.1% and 35.4%, respectively) (Fig. 5d, e, f). Additionally, we observed that plants co-inoculated with *C. blakesleeana* and *M. perniciosa* increased the content of Chl *a* (59.1%), Chl *b* (58.3%), and carotenoids (61.4%) compared to plants infected only with the pathogen (Fig. 5c, d, f).

## Discussion

This study aimed to compare the diversity and composition of the endophytic fungal community isolated from the leaves and stems of *Theobroma grandiflorum* in Southwestern Amazon. Additionally, we evaluated the potential of these endophytic fungi to control the pathogen *Moniliophthora perniciosa* and to promote the growth of *T. grandiflorum*. Our results indicate that *T. grandiflorum* trees harbor a high diversity of culturable endophytic fungi, belonging to 23 known genera, and the majority derives from the phylum Ascomycota (90.6%). Previous studies using culture-dependent methods have also found that Amazonian plants, such as *Hevea brasiliensis* [55], *Euterpe precatoria* [56], as well as plants from other regions, such as *Colobanthus quitensis* in Antarctica [57] and bamboo in China [58], predominantly contain fungal isolates classified within Ascomycota. This predominance is often attributed to the use of standard isolation methods, particularly using PDA medium [59]. In our study, we isolated a total of 481 fungi from the leaves and stems of *T. grandiflorum*. This contrasts with [60], who recovered 13 endophytic fungi from *T. grandiflorum* trees in a different region of the Amazon (state of Amazonas) using PDA medium. As indicated by [61], the differences in results may be related to the number and geographic location of the trees sampled, as well as the composition of the culture media used for isolation. For example, in the present study different media were used as nutrient sources, such as PDA and OA, whereas [60] used only PDA for the isolation of endophytic fungi.

Regarding relative abundance, our findings align with other studies indicating that plants in natural systems host endophytic communities with numerous taxa with low relative abundance, dominated by a few fungal taxa [61, 62]. In our study, *Diaporthe* and *Lasiodiplodia* were the most abundant genera in both leaves and stems. However, several genera had low relative abundance and were exclusive to specific tissues. For instance, *Hypoxylon*, *Nigrospora*, *Geotrichum*, *Guignardia*, and *Cunninghamella* were found in leaf tissue, while *Coprinellus*, *Gelasinospora*, *Daldinia*, *Myrmecridium*, *Trichoderma*, *Curvularia*, and *Endomelanconiopsis* were exclusive to the stem. Genera such as *Colletotrichum*, *Penicillium*, *Curvularia*, *Fusarium*, *Talaromyces*, and *Clonostachys*, which were shared between tissues and had low relative abundance, were also recovered from *Theobroma cacao* in the Amazon [60], a closely related species to *T. grandiflorum*. This pattern supports the concept that the presence of diverse fungal genera at low abundance increases competitive interactions, thereby preventing any single species from becoming overly dominant and potentially causing disease [63].

Despite the anatomical and functional differences between leaves and stems, our analysis revealed no significant differences in the richness, diversity, or composition of the endophytic fungal communities in *T. grandiflorum*. This finding suggests that the host plant may exert a stronger influence on its endophytic microbiome as a whole than the specific tissue type alone. This pattern aligns with observations in other tropical species, such as *H. brasiliensis* [64], and underscores the idea that broad host-specific factors can sometimes override the effect of tissue specificity. Ultimately, this reinforces the notion that the endophytic communities is a complex process, where plant-related factors (e.g., plant genotype and immune responses) may play a more defining role than individual tissue niches, especially when compared to external drivers like geographic location, management practices, and climate, which are known to be major influencers of fungal community structure [56, 65, 66].

Endophytic microorganisms, particularly fungi, play a significant role in disease control and promotion of plant growth [67]. These fungi produce bioactive molecules and enzymes that fight phytopathogens and enhance nutrient absorption by the host [68]. Our study revealed that the 14 endophytic fungi isolated from *T. grandiflorum* demonstrated versatility in the production of hydrolytic enzymes in culture media. The fact that amylolytic, cellulolytic, and proteolytic activities were commonly observed among these fungi may be related to their role in colonizing the host plant [69]. *Clonostachys rosea*, *C. pseudochroleuca*, *C. fructicola*, and *Daldinia* species produced amylase, cellulase, and protease, enzymes associated with plant cell wall degradation. In particular, cellulase and protease enzymes also constitute key mechanisms in mycoparasitism. Additionally, these enzymes, along with lipase, may indirectly reduce pathogens by degrading fungal cell wall and plasma membrane [70]. However, our results showed that only *G. calospora*, *T. tawa*, and *Cladosporium* sp. exhibited significant lipolytic activity. Similarly, only a few endophytic fungi from açaí palms exhibited lipolytic activity in solid media [71].

This potential link between enzyme production and biocontrol is supported by our i*n vitro* antagonism results. The fungi from the genera *Trichoderma* (*T. orientale* and *T. tawa*) and *C. blakesleeana* showed the best results, with a pathogen growth inhibition rate close to 65%. Notably, these genera are known to be prolific producers of hydrolytic enzymes. For instance, the strong inhibitory activity of the *Trichoderma* isolates, for instance, aligns well with their recognized capacity for enzyme-mediated mycoparasitism [28]. This result is comparable to a study that analyzed the potential of *Trichoderma* species in suppressing *M. roreri* in cacao trees, achieving up to 68.8% suppression [72]. Furthermore, different isolates of *T. harzianum* showed high levels of mycoparasitism, antibiosis, and potential antagonism to *M. roreri* [73]. On the other hand, there are no previous reports of *C. blakesleeana* as a biocontrol agent of phytopathogens, but it has shown promise in the health sector, such as its ability to degrade cholesterol [74] and its potential for the microbial transformation of the contraceptive drug etonogestrel into new metabolites [75].

Interestingly, the results of the screening of endophytic fungi in cupuaçu seedlings, conducted in a greenhouse, revealed that the isolates *T. tawa* and *C. blakesleeana* showed promising results in controlling *M. perniciosa*, as observed in the in vitro antagonism test. In the second experiment, *T. tawa* and *C. blakesleeana* reduced the incidence of witches’ broom in cupuaçu seedlings by 50% and 70%, respectively. This is the first report in the literature of the endophytic fungus *C. blakesleeana* successfully controlling a plant pathogen. While fungi of the genus *Trichoderma* are widely recognized as leading biocontrol agents against phytopathogens [76, 77], this discovery underscores the untapped potential of the Amazon’s vast biodiversity. It paves the way for exploring new and innovative biological control strategies, addressing critical challenges in plant disease management and advancing sustainable agriculture. Field experiments have also demonstrated the ability of *T. stromaticum* isolates to reduce the incidence of *M. perniciosa* in cacao witches’ brooms [78]. However, this study was conducted in the Atlantic Forest biome, which presents climatic conditions distinct from those of the Amazon. This highlights the importance of conducting research on fungal biocontrol agents specifically within the Amazon region, where unique environmental conditions and biodiversity may influence the effectiveness and adaptation of these organisms in managing plant pathogens. Additionally, in the Atlantic Forest, the endophytic fungus *Gliocladium catenulatum* also reduced the incidence of this disease in cacao seedlings in a greenhouse by 70% [26].

Remarkably, *T. tawa* and *C. blakesleeana* promoted the growth of cupuaçu plants, increasing the height and dry weight of the aerial parts. These data may be associated with the increase in photosynthetic pigment content that these fungi caused in the plants. The increase in chlorophyll and carotenoid concentrations by endophytic fungi leads to an incremental increase in the photosynthesis rate, which is followed by higher plant growth [79]. In contrast, our data revealed that in plants inoculated only with *M. perniciosa*, the chlorophyll a and b contents decreased. The infection of plants by fungal pathogens affects the plant photosynthetic pathway by decreasing the activity of mesophyll cells and ribulose-1,5-bisphosphate carboxylase/oxygenase (Rubisco), along with disrupting stomatal regulation during transpiration [80]. However, in this study, in the coinoculation of *C. blakesleeana* and *M. perniciosa*, we observed an increase in the contents of Chl *a* and *b*, and carotenoids. These results may be associated with the fact that endophytic fungi antagonistic to phytopathogens are capable of enhancing photosynthesis by inducing the upregulation of genes and pigments, as well as activating biochemical pathways that mitigate the damage caused by pathogen-induced reactive oxygen species (ROS) [81].

Although our findings demonstrate the potential of endophytic fungi, particularly T. *tawa* and *C. blakesleeana*, in controlling *M. perniciosa* and promoting the growth of *T. grandiflorum* under greenhouse conditions, an important limitation of this study is the lack of field validation. Therefore, future studies should focus on field trials to evaluate the efficacy, persistence, and adaptability of these endophytic fungi under real cultivation conditions in the Amazon. Such research will be crucial to confirm their practical applicability as biocontrol agents and to advance sustainable disease management strategies for cupuaçu.

## Conclusions

Our results reveal that *Theobroma grandiflorum* harbors a diversity of endophytic fungal genera with low relative abundance, including several fungi antagonistic to *Moniliophthora perniciosa*. In this study, the endophytic fungi *Trichoderma tawa* and *Cunninghamella blakesleeana* demonstrated high antagonistic activity in vitro against the pathogen, as well as strong capacity to control *M. perniciosa* in cupuaçu plants. Additionally, these fungi promoted the growth of cupuaçu seedlings, highlighting their potential both as biocontrol agents and plant growth promoters. These findings indicate *T. tawa* and *C. blakesleeana* as promising candidates for the integrated management of *M. perniciosa*. Future studies should focus on the large-scale application of these fungal isolates and the evaluation of their effectiveness in agroforestry systems under field conditions, contributing to the sustainable management of cupuaçu cultivation in the Amazon.

## Supplementary Information

Below is the link to the electronic supplementary material.

Supplementary Material 1
