# Supplementary material for: Diversity of Endophytic Fungi in Theobroma grandiflorum and their Potential for Biological Control of Witches’ Broom Disease and Promotion of Cupuaçu Seedling Growth in the Amazon
Source: Curr Microbiol. 2026 Apr 3;83(5):280. doi: 10.1007/s00284-026-04853-x (PMC13048954; doi:10.1007/s00284-026-04853-x)
Supplement: Supplementary file 1 — Supplementary Material 1 [file 284_2026_4853_MOESM1_ESM.docx]

**Current Microbiology**

Supporting materials

**Diversity of endophytic fungi in *Theobroma grandiflorum* and their potential for biological control of witches' broom disease and promotion of cupuaçu seedling growth in the amazon**

Jusley Souza Santos ^1‡^. Nárcya Trindade de Souza ^1‡^. Thalya da Silva Rodrigues ^1‡^. Fernando José Fernandes Martins Junior ^1^. Laryssa dos Santos Prado ^1^. Fernanda Viana Diniz ^1^. Erlangela Rocha Viga ^1^. [Berenice Kussumoto de Alcântara da Silva](https://www.escavador.com/sobre/3402044/berenice-kussumoto-de-alcantara) ^1^. Clarice Maia Carvalho ^1^. Leila Priscila Peters ^2*^

^1^ Centro de Ciências Biológicas e da Natureza. Universidade Federal do Acre. Rio Branco. Acre. Brazil.

^2^ Centro de Ciências da Saúde e do Desporto. Universidade Federal do Acre. Rio Branco. Acre. Brazil.

^‡^Jusley Souza Santos. Nárcya Trindade de Souza and Thalya da Silva Rodrigues contributed equally to this work and share first authorship.

*Corresponding author: [leila.peters@ufac.br](mailto:leila.peters@ufac.br) (Peters. L.P)

**Table of contents**

| N^o^ | Figure Capture | Page |
| --- | --- | --- |
| 1 | **Fig. S1** Sampling sites for leaf and stem samples of *Theobroma grandiflorum*. **a** Collection sites in the southwestern Amazon. **b** Healthy adult cupuaçu tree located at the forest edge (sampling example) | 2 |
| 2 | **Fig. S2** Colony of endophytic fungi isolated from the leaf and stem of *Theobroma grandiflorum*. The fungi were cultivated in PDA medium for 10 days at 28ºC | 3 |
| 3 | **Table S1** Plant growth parameters and disease incidence (%) in cupuaçu plants inoculated with each of the 14 fungal isolates, with and without *Moniliophthora perniciosa*, under greenhouse conditions. The letter E denotes cupuaçu plants inoculated solely with the endophytic fungus, while the letter P indicates cupuaçu plants co-inoculated with the endophytic fungus and the pathogen | 4 |

**Fig. S1** Sampling sites for leaf and stem samples of *Theobroma grandiflorum*. **a** Collection sites in the southwestern Amazon. **b** Healthy adult cupuaçu tree located at the forest edge (sampling example).


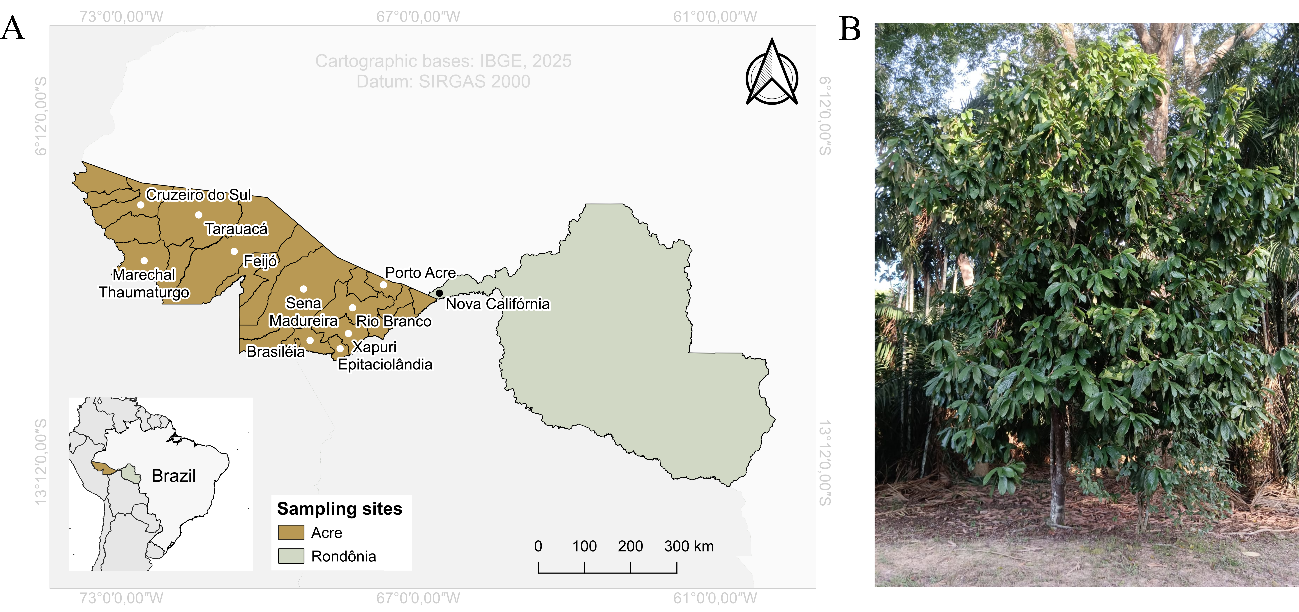


**Fig. S2** Colony of endophytic fungi isolated from the leaf and stem of *Theobroma grandiflorum*. The fungi were cultivated in PDA medium for 10 days at 28ºC.


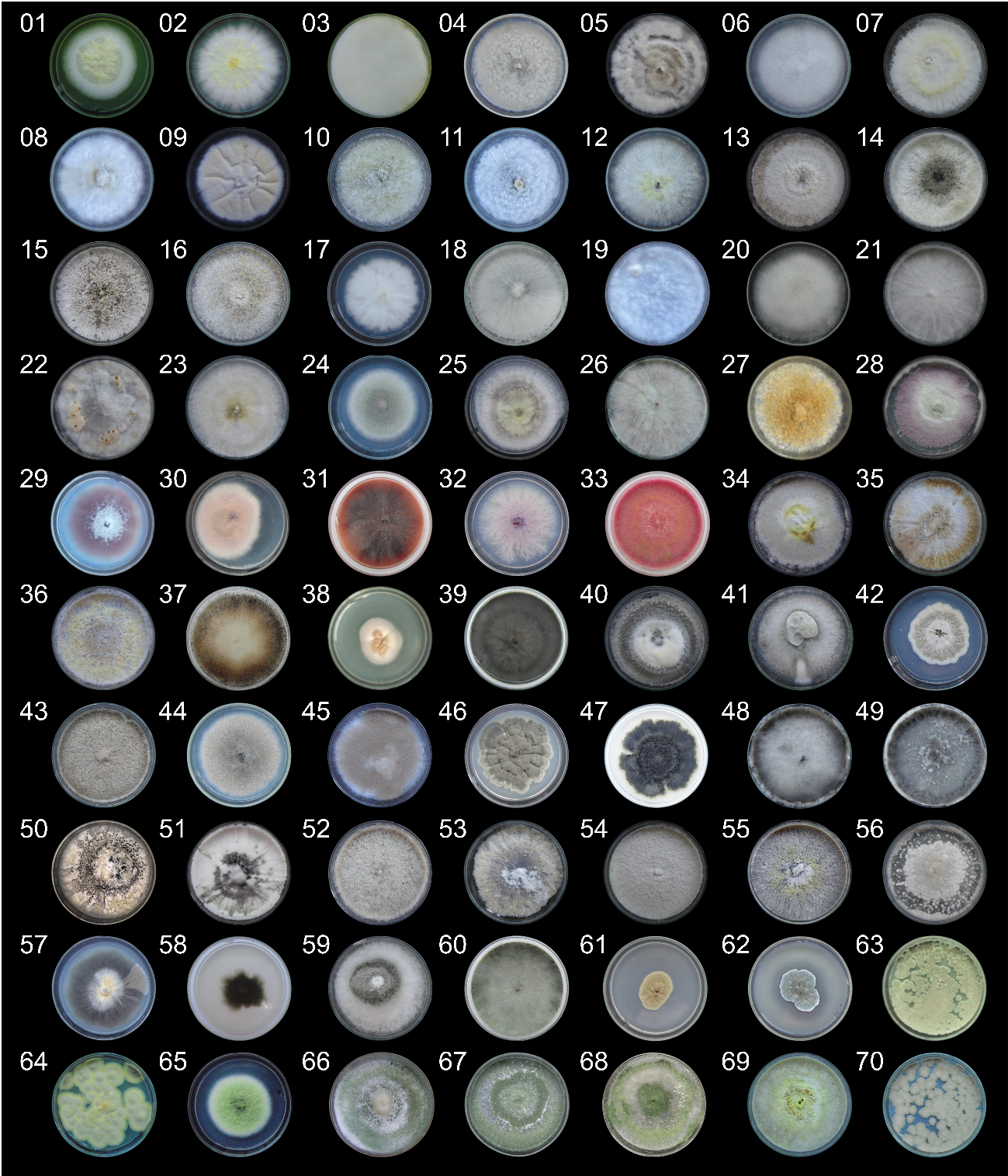


**Table S1** Plant growth parameters and disease incidence (%) in cupuaçu plants inoculated with each of the 14 fungal isolates, with and without *Moniliophthora perniciosa*, under greenhouse conditions. The letter E denotes cupuaçu plants inoculated solely with the endophytic fungus, while the letter P indicates cupuaçu plants co-inoculated with the endophytic fungus and the pathogen.

| Endophytic fungus | Treatment | Height | Shoot | Root | Disease incidence (%) |
| --- | --- | --- | --- | --- | --- |
| *Trichoderma tawa* | E | 22.42 ± 2.08 ^ns^ | 33.71 ± 3.60 ^abc^ | 18.50 ± 3.10 ^ns^ | 0.00 ± 0.00 ^d^ |
|  | E+P | 20.03 ± 2.78 ^ns^ | 34.95 ± 3.72 ^abc^ | 21.90 ± 2.99 ^ns^ | 0.00 ± 0.00 ^d^ |
| *Daldinia* sp. | E | 22.92 ± 1.46 ^ns^ | 35.12 ± 5.52 ^abc^ | 19.87 ± 3.97 ^ns^ | 0.00 ± 0.00 ^d^ |
|  | E+P | 20.58 ± 2.08 ^ns^ | 39.45 ± 3.86 ^a^ | 20.82 ± 3.15 ^ns^ | 0.00 ± 0.00 ^d^ |
| *Cladosporium* sp. | E | 21.18 ± 3.02 ^ns^ | 28.37 ± 7.61 ^c^ | 17.56 ± 6.17 ^ns^ | 0.00 ± 0.00 ^d^ |
|  | E+P | 19.33 ± 4.24 ^ns^ | 33.14 ± 3.38 ^abc^ | 19.52 ± 1.99 ^ns^ | 67 ± 0.52 ^abc^ |
| *Colletotrichum fructicola* | E | 21.14 ± 3.02 ^ns^ | 31.34 ± 3.04 ^abc^ | 18.11 ± 2.98 ^ns^ | 0.00 ± 0.00 ^d^ |
|  | E+P | 22.25 ± 2.82 ^ns^ | 33.17 ± 5.85 ^abc^ | 15.79 ± 4.77 ^ns^ | 100 ± 0.00 ^a^ |
| *Endomelanconiopsis microspora* | E | 21.52 ± 2.54 ^ns^ | 30.12 ± 5.13 ^bc^ | 18.56 ± 2.50 ^ns^ | 0.00 ± 0.00 ^d^ |
|  | E+P | 22.13 ± 2.62 ^ns^ | 35.80 ± 2.32 ^abc^ | 18.96 ± 2.56 ^ns^ | 67 ± 0.52 ^abc^ |
| *Talaromyces pinophilus* | E | 19.93 ± 2.89 ^ns^ | 34.51 ± 4.77 ^abc^ | 19.13 ± 1.78 ^ns^ | 0.00 ± 0.00 ^d^ |
|  | E+P | 19.80 ± 3.62 ^ns^ | 31.08 ± 4.05 ^abc^ | 15.48 ± 4.12 ^ns^ | 17 ± 0.41 ^cd^ |
| *Cunninghamella blakesleeana* | E | 21.47 ± 2.28 ^ns^ | 35.65 ± 2.06 ^abc^ | 19.57 ± 1.36 ^ns^ | 0.00 ± 0.00 ^d^ |
|  | E+P | 22.17 ± 2.46 ^ns^ | 39.31 ± 2.64 ^a^ | 20.15 ± 1.87 ^ns^ | 0.00 ± 0.00 ^d^ |
| *Myrmecridium* sp. | E | 19.83 ± 1.75 ^ns^ | 34.79 ± 3.38 ^abc^ | 19.76 ± 1.57 ^ns^ | 0.00 ± 0.00 ^d^ |
|  | E+P | 21.08 ± 3.06 ^ns^ | 33.44 ± 3.99 ^abc^ | 18.80 ± 4.25 ^ns^ | 17 ± 0.41 ^cd^ |
| *Clonostachys pseudochroleuca* | E | 23.07 ± 4.85 ^ns^ | 33.62 ± 4.05 ^abc^ | 17.73 ± 2.61 ^ns^ | 0.00 ± 0.00 ^d^ |
|  | E+P | 19.87 ± 3.47 ^ns^ | 31.95 ± 4.06 ^abc^ | 18.04 ± 4.23 ^ns^ | 67 ± 0.52 ^abc^ |
| *Coprinellus radians* | E | 19.12 ± 1.90 ^ns^ | 31.47 ± 2.57 ^abc^ | 16.86 ± 2.29 ^ns^ | 0.00 ± 0.00 ^d^ |
|  | E+P | 21.87 ± 2.33 ^ns^ | 34.69 ± 3.65 ^abc^ | 18.05 ± 3.64 ^ns^ | 50 ± 0.55 ^abcd^ |
| *Clonostachys rosea* | E | 20.55 ± 2.48 ^ns^ | 34.07 ± 4.04 ^abc^ | 18.64 ± 2.65 ^ns^ | 0.00 ± 0.00 ^d^ |
|  | E+P | 21.83 ± 2.77 ^ns^ | 34.11 ± 3.83 ^abc^ | 18.28 ± 1.93 ^ns^ | 33 ± 0.52 ^bcd^ |
| *Gelasinospora calospora* | E | 21.47 ± 3.00 ^ns^ | 31.70 ± 4.74 ^abc^ | 18.29 ± 4.14 ^ns^ | 0.00 ± 0.00 ^d^ |
|  | E+P | 21.13 ± 1.64 ^ns^ | 36.11 ± 5.41 ^abc^ | 20.83 ± 1.70 ^ns^ | 83 ± 0.41 ^ab^ |
| *Talaromyces purpureogenus* | E | 21.08 ± 3.31 ^ns^ | 28.31 ± 3.79 ^c^ | 17.67 ± 2.30 ^ns^ | 0.00 ± 0.00 ^d^ |
|  | E+P | 22.25 ± 2.82 ^ns^ | 35.90 ± 3.32 ^abc^ | 18.17 ± 2.10 ^ns^ | 17 ± 0.41 ^cd^ |
| *Trichoderma orientale* | E | 22.12 ± 2.61 ^ns^ | 28.83 ± 2.77 ^bc^ | 18.34 ± 3.18 ^ns^ | 0.00 ± 0.00 ^d^ |
|  | E+P | 20.25 ± 2.70 ^ns^ | 33.59 ± 4.12 ^abc^ | 18.40 ± 1.81 ^ns^ | 67 ± 0.52 ^abc^ |
| Control | Positive | 17.70 ± 1.19 ^ns^ | 36.61 ± 4.14 ^abc^ | 18.57 ± 1.36 ^ns^ | 100 ± 0.00 ^a^ |
|  | Negative | 19.58 ± 1.50 ^ns^ | 37.53 ± 3.15 ^ab^ | 20.16 ± 2.01 ^ns^ | 0.00 ± 0.00 ^d^ |

Values with different letters in the same column are significantly different (p < 0.05), according to one-way analysis of variance (ANOVA) followed by Tukey’s test. Data are means ± SD. “ns” indicates non-significant differences.
